# Data on the absorbance of glucose during the acid hydrolysis of the sugarcane bagasse

**DOI:** 10.1016/j.dib.2019.103894

**Published:** 2019-04-03

**Authors:** Abbas F.M. Alkarkhi, Wasin A.A. Alqaraghuli, Yusri Yusup, Salem S. Abu Amr, M.N. Mahmud, Nugroho Dewayantoa

**Affiliations:** a Universiti Kuala Lumpur, Malaysian Institute of Chemical & Bioengineering Technology (UniKL, MICET), 78000, Melaka, Malaysia; b Skill Education Center, PA, A-07-03 Pearl Avenue, Sungai Chua, 43000 Kajang, Selangor, Malaysia; c Environmental Technology, School of Industrial Technology, Universiti Sains Malaysia, 11800 Pulau Pinang, Malaysia

## Abstract

This article presents data relating to the changes in absorbance of glucose during the acid hydrolysis of sugarcane bagasse using sulphuric acid. This dataset also contains the moisture content, volatile matter, and fixed carbon of the sugarcane bagasse. The results of the analysis of variance (ANOVA) and the interaction plots between reaction time, temperature, and ratio are also presented. The data revealed that absorbance of glucose is increasing by increasing the temperature and time. Moreover, the best ratio for the highest absorbance of glucose was achieved at 1:20.

**Table:** Specifications table

Subject area	Organic chemistry
More specific	Physical organic chemistry
Subject area	
Type of data	Table and figure
How data was acquired	Electronic balance and ultraviolet–visible spectrophotometry
Data format	Experimental data and analysis
Experimental factors	The sample was cleaned, dried, cut, and ground
Experimental features	Different settings of time, temperature, and sugarcane bagasse to sulphuric acid ratio
Data Source Location	University of Kuala Lumpur, Malaysian Institute of Chemical and Bio Engineering Technology (UniKL- MICET), Melaka, Malaysia.
Data accessibility Related research article	Within this article B.P. Lavarack, G.J.Grin, D. Rodman The acid hydrolysis of sugarcane bagasse hemicellulose to produce xylose, arabinose, glucose and other products, Biomass and Bioenergy, 23 (2002) 367–380 [1]

**Table:** 

**Value of the data** • The data is important for the analysis of the utilization of sugarcane bagasse to produce glucose using the acid hydrolysis process in the agricultural waste sector. • Common independent process parameters: reaction time, temperature and acid ratio in relation to glucose are presented.

## 1 Data

**Fig. 1:**
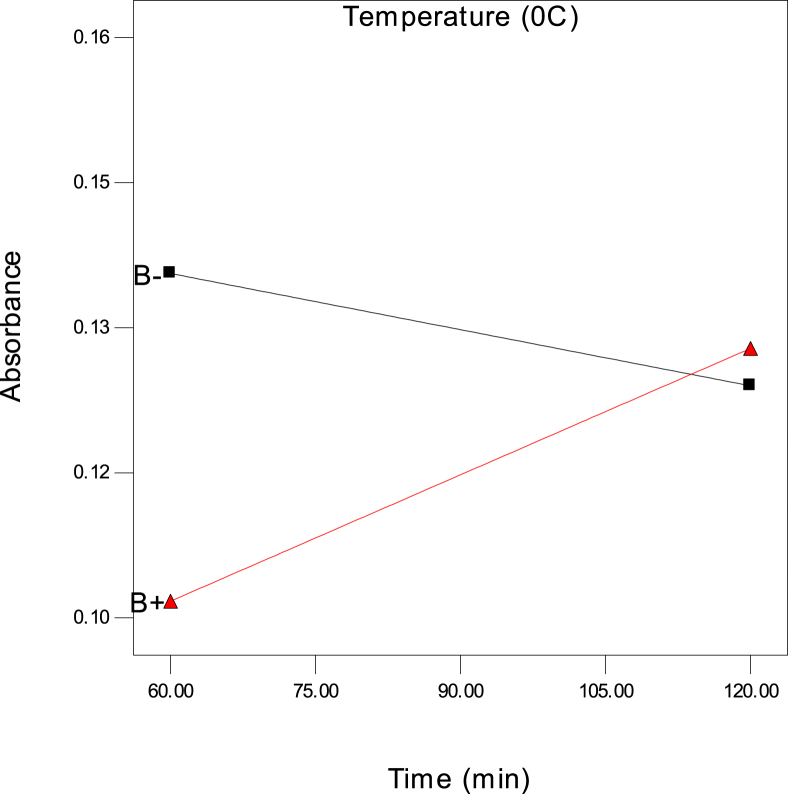
Two-factor interaction showing the behaviour of time (t) and temperature (T) (■30 ▲60) on the absorbance of glucose at the ratio 1:10.

**Fig. 2:**
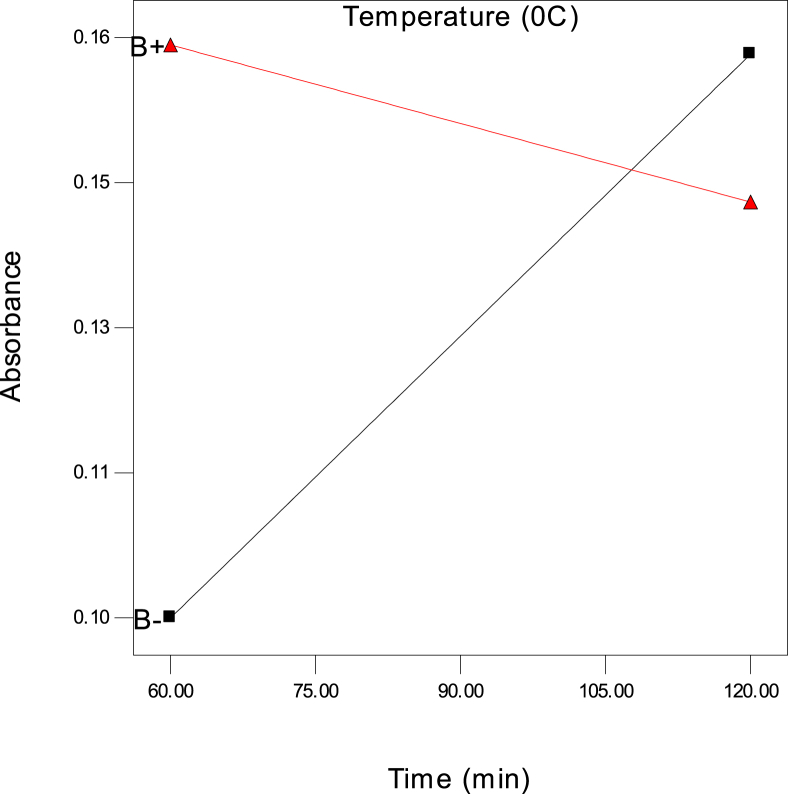
Two-factor interaction showing the behaviour of time (t) and temperature (T) (■30 ▲60) on the absorbance of glucose at a ratio 1:20.

The data in this article includes the absorbance of glucose during the acid hydrolysis of sugarcane bagasse covering all possible combination of three variables, time (t), temperature (T), and ratio as listed in Table 1 (Two-level factorial design augmented by centre points). This dataset also includes the moisture content (Table 2), volatile matter (Table 3), and fixed carbon (Table 4) of the sugarcane bagasse. The results of the analysis of variance (ANOVA) are also listed (Table 5). All of the experimental filtered data were presented in the form of a table. The interaction plots are shown in the form of figures (Fig. 1, Fig. 2) for the behaviour of the different variables in the presence of the variables towards the output or response. The optimum setting of time, temperature, and ratio for the absorbance of glucose is given in Table 6.

**Table 1:** The two-level factorial design of the absorbance of glucose using the acid hydrolysis process; the design was augmented with four runs at the centre for three factors, time (t), temperature, and ratio of sugarcane bagasse.

Time (min)	Temperature (^0^C)	Ratio	Absorbance
120	30	1:20	0.161
60	60	1:20	0.162
120	30	1:10	0.124
60	30	1:20	0.100
120	60	1:20	0.145
90	45	1:20	0.155
90	45	1:10	0.140
60	30	1:10	0.136
90	45	1:20	0.155
90	45	1:10	0.140
60	60	1:10	0.101
120	60	1:10	0.128

**Table 2:** Moisture content (%) of the sugarcane bagasse.

Sample	Weight before drying (g)	Weight on petri dish before drying (g)	Weight on petri dish after drying (g)	Weight after drying (g)	Moisture content (%)
1	5	73.60	73.16	4.56	8.8
2	5	72.14	71.67	4.53	9.4
3	5	73.23	72.75	4.52	9.6

**Table 3:** Volatile matter (%) of the sugarcane bagasse.

Samples	Weight before drying (g)	Weight on petri dish before drying (g)	Weight on petri dish after drying (g)	Weight after drying (g)	Moisture content (%)
1	5	73.16	71.12	2.96	40.8
2	5	71.67	69.56	2.89	42.2
3	5	72.75	70.67	2.92	41.6

**Table 4:** Ash value (%) of the sugarcane bagasse.

Samples	Weight before drying (g)	Weight on petri dish before drying (g)	Weight on petri dish after drying (g)	Weight after drying (g)	Moisture content (%)
**1**	5	71.12	70.33	4.21	15.8
**2**	5	69.56	68.76	4.20	16.0
**3**	5	70.67	69.9	4.23	15.4

**Table 5:** The results of the analysis of variance (ANOVA) for the absorbance of glucose.

Source	Sum of squares	DF	Sum of Mean	F-Value	p-value
Model	4.201E-003	7	6.001E-004	119.69	0.0012
t	4.35E-04	1	4.35E-04	86.78	0.0026
T	2.81E-05	1	2.81E-05	5.61	0.0986
ratio	9.90E-04	1	9.90E-04	197.47	0.0008
t*T	1.90E-04	1	1.90E-04	37.92	0.0086
t*ratio	1.05E-04	1	1.05E-04	20.97	0.0196
T*ratio	7.41E-04	1	7.41E-04	147.81	0.0012
t*T*ratio	1.71E-03	1	1.71E-03	341.28	0.0003
Residual	1.504E-005	3	5.01E-06		
Total	4.846E-003	11

**Table 6:** The optimum setting of the selected variables to produce maximum glucose absorbance.

Time	Temperature	Ratio	Absorbance
60	60	2	0.162
120	30	2	0.161

## 2 Experimental design, materials, and methods

Experiments were performed to collect the data. The reagent and raw material used are sugarcane bagasse, sulphuric acid, and distilled water. The apparatus used are the conical flask, Erlenmeyer flask, oven, filter paper, blender, water bath, furnace, beakers, measuring cylinder, and ultraviolet–visible spectroscopy or ultraviolet–visible spectrophotometry (also referred to as UV–Vis or UV/Vis).

Sugarcane bagasse is used in this study. Sugarcane bagasse obtained according to the quantity needed for the project. Physical pre-treatment of sugarcane bagasse was done, such as cleaning, drying, cutting and grounding. Then, the small volume of air dried sugarcane bagasse was dried in an oven at the temperature range 50–60 °C for 20 h. A mass of 10 g oven dried sugarcane bagasse was collected and fixed throughout the experiments. The mass ratios of sulphuric acid were chosen to be 1:10 and 1:20 (grams of the sugarcane bagasse/mL of sulphuric acid solution). For 1:10, 100 mL of sulphuric acid used was applied, while for 1:20, 200 mL was applied to the sugarcane bagasse in a 250-mL Erlenmeyer flask. Distilled water was used to top-up the mixture to the final volume of 250 mL. The Erlenmeyer flask was then put in a water bath at different temperatures (30, 45, and 60 °C) and time (60, 90, and 120 min) for the acid hydrolysis process to occur. After the run is complete, the sample was filtered and diluted in a 100 mL volumetric flask. The absorbance of glucose was measured using the ultraviolet–visible spectroscopy.

The drying oven was used to determine the moisture content of the sugarcane bagasse. Before drying, the 5 g of the sugarcane bagasse was ground using a blender. The fixed carbon (FC) is determined by subtracting the moisture, volatile matter, and ash percentages from the sample. The sample was dried at 100 °C for five to 6 h. The sample was then re-weighed to obtain the final mass. Moisture content (%) is calculated from the difference between the wet sample and the dried sample. Volatile matter (VM) and ash value (AV) in percentage (excluding water vapour) was measured by weighing the initial and final mass of the sugarcane bagasse after placing it in the furnace at 500 °C for an hour. Then the FC calculated following Eq. (1) [2].

(1) Fixedcarbon(FC)(%)=100−moisture(%)+volatilematter(%)+ashvalue(%)

**Fig. 3:**
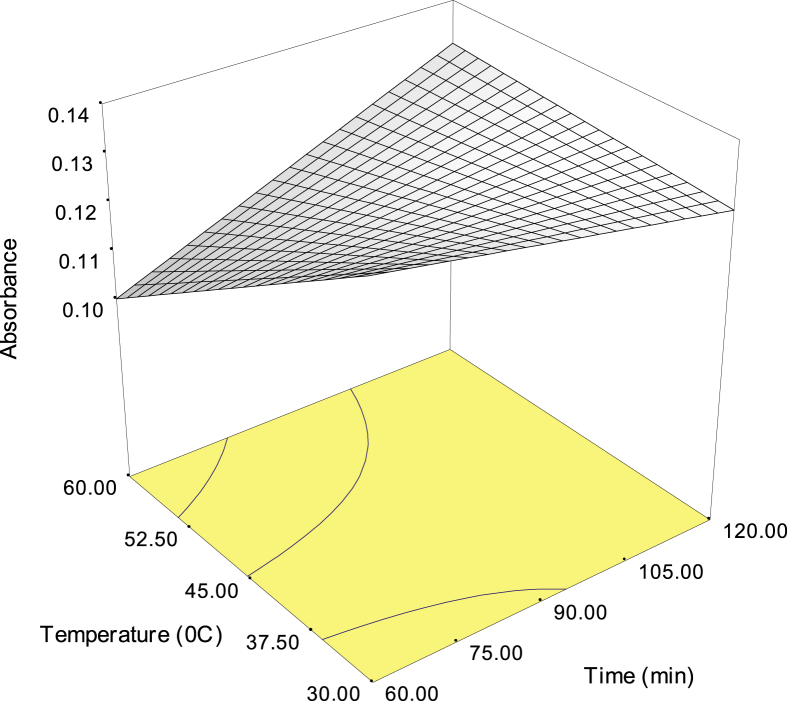
Three-dimensional response surface plot showing the behaviour of time (t) and temperature (T) on the absorbance of glucose at a ratio 1:10.

**Fig. 4:**
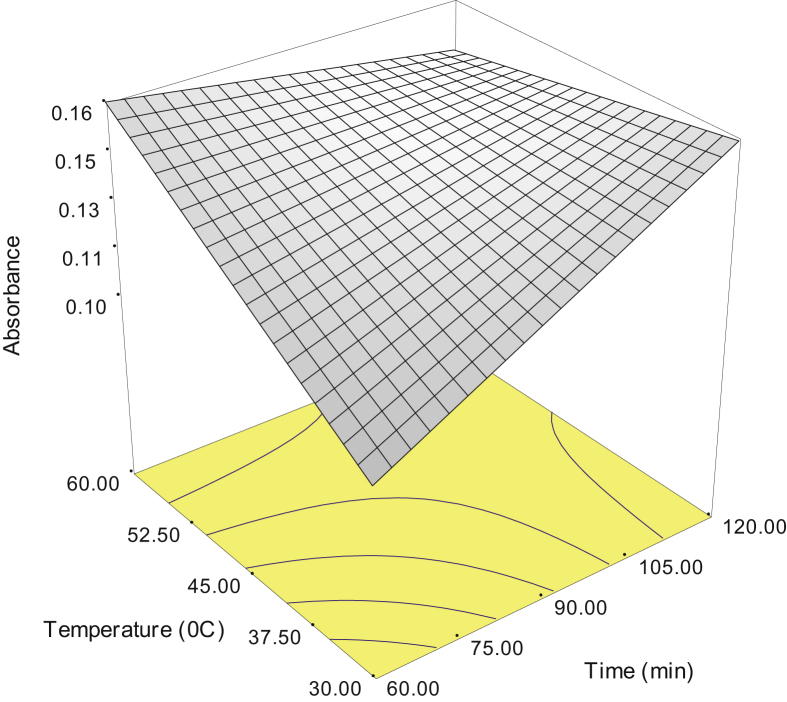
Three-dimensional response surface plot showing the behaviour of time (t) and temperature (T) on the absorbance of glucose at a ratio 1:20.

The data were analysed using analysis of variance (ANOVA) [3], [4] as shown in Table 5. The behaviour of significant interactions is presented in Fig. 1, Fig. 2. Three-dimensional response surface plots are given in Fig. 3, Fig. 4 showing the behaviour of the response (absorbance of glucose) at all possible combination of time and temperature with the ratio.

The maximum absorbance of glucose (0.16) was achieved by setting the time (t), temperature (T) and maintaining the ratio 1:20 as listed in Table 6.
