# Supplementary material for: Data on the absorbance of glucose during the acid hydrolysis of the sugarcane bagasse
Source: Data Brief. 2019 Apr 3;24:103894. doi: 10.1016/j.dib.2019.103894 (PMC6461596; doi:10.1016/j.dib.2019.103894)
Supplement: Multimedia component 1 [file mmc1.doc]

Conflict of Interest and Authorship Conformation Form

Please check the following as appropriate:

- All authors have participated in (a) conception and design, or analysis and interpretation of the data; (b) drafting the article or revising it critically for important intellectual content; and (c) approval of the final version.
- This manuscript has not been submitted to, nor is under review at, another journal or other publishing venue.
- The authors have no affiliation with any organization with a direct or indirect financial interest in the subject matter discussed in the manuscript
- The following authors have affiliations with organizations with direct or indirect financial interest in the subject matter discussed in the manuscript:

Author’s name Affiliation

Abbas F.M. Alkarkhi, Universiti Kuala Lumpur, Malaysian Institute of Chemical & Bioengineering Technology (UniKL, MICET), 78000, Melaka, Malaysia.

Wasin A.A. Alqaraghuli, Skill Education center, PA, A-07-03 Pearl Avenue, Sungai Chua, 43000 Kajang, Selangor, Malaysia.

Yusri Yusup, Environmental Technology, School of Industrial Technology, Universiti Sains Malaysia, 11800 Pulau Pinang, Malaysia.

Salem S. Abu Amr, Universiti Kuala Lumpur, Malaysian Institute of Chemical & Bioengineering Technology (UniKL, MICET), 78000, Melaka, Malaysia.

Nugroho Dewayantoa Universiti Kuala Lumpur, Malaysian Institute of Chemical & Bioengineering Technology (UniKL, MICET), 78000, Melaka, Malaysia.

M.N. Mahmud, Universiti Kuala Lumpur, Malaysian Institute of Chemical & Bioengineering Technology (UniKL, MICET), 78000, Melaka, Malaysia.
